# “Heart Appearance” Infarction of the Pons: A Case Report

**DOI:** 10.1155/2012/690903

**Published:** 2012-10-09

**Authors:** Keisuke Ishizawa, Mikiko Ninomiya, Yoshihiko Nakazato, Toshimasa Yamamoto, Nobuo Araki

**Affiliations:** Department of Neurology, Faculty of Medicine, Saitama Medical University, Morohongo 38, Moroyama, Iruma-gun, Saitama, 350-0495, Japan

## Abstract

“Heart appearance” on magnetic resonance imaging (MRI) is a unique presentation of bilateral medial medullary infarction. In contrast, “heart appearance” infarction of the pons has rarely been featured in the medical literature. In this paper, we present a case of “heart appearance” infarction of the pons with its MRI and magnetic resonance angiography (MRA) findings. The patient was an 87-year-old male who manifested with weakness in the four extremities. Later, bulbar palsy and tetraplegia became apparent, and he eventually was trapped in locked-in syndrome. Brain MRI disclosed a “heart appearance” lesion in the pons, which was high on diffusion-weighted image MRI and low on apparent diffusion coefficient map MRI. Brain MRA demonstrated that the basilar artery remained intact. A diagnosis of fresh, bilateral pontine infarction with a “heart appearance” was made. After the treatment he was transferred to another hospital for long-term care. This case suggests that bilateral ischemic involvement of the pons is possible even in the context of an intact basilar artery.

## 1. Introduction

“Heart appearance” on magnetic resonance imaging (MRI) is a unique presentation of bilateral medial medullary infarction [1–3]. In contrast, “heart appearance” infarction of the pons has rarely been featured in the medical literature. In this paper, we present a patient affected with “heart appearance” infarction of the pons with the MRI and magnetic resonance angiography (MRA) findings. 

## 2. Case Presentation

**Figure 1:**
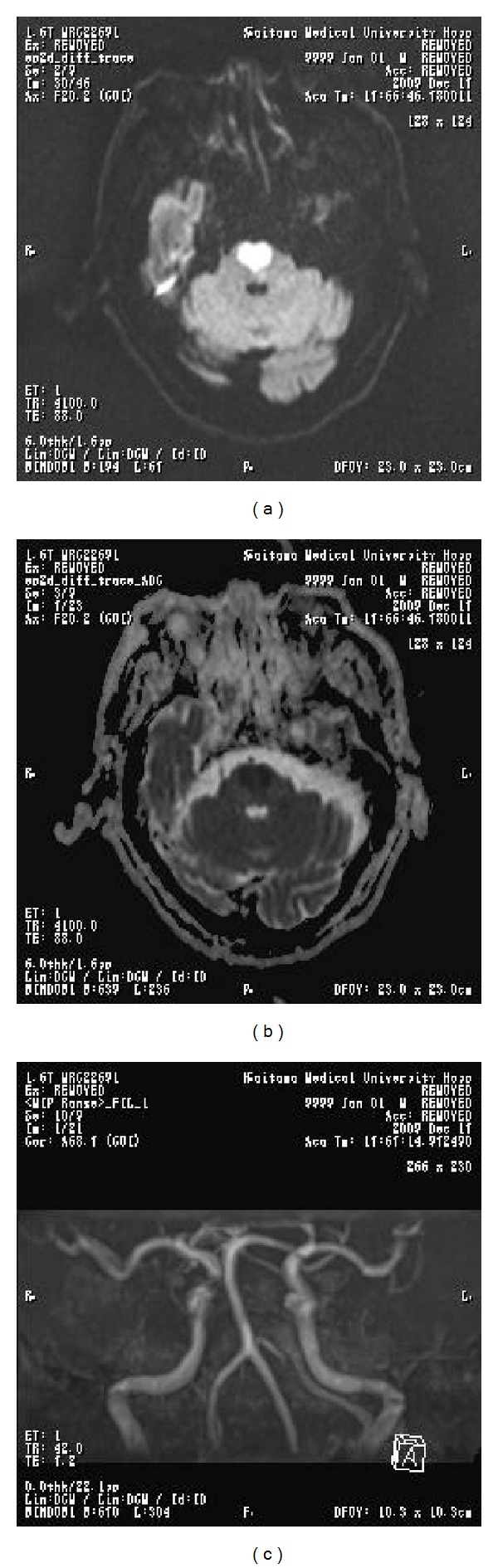
(a) The brain diffusion-weighted image MRI (DWI) of the patient is shown. Note the “heart appearance” lesion showing high intensity in the pontine base. (b) The brain apparent diffusion coefficient map MRI (ADC) of the patient is shown. The “heart appearance” lesion shows low intensity on ADC, indicating, in conjunction with the finding on DWI, that the lesion is a fresh infarct. (c) The brain magnetic resonance angiography (MRA) of the patient is shown. The basilar artery remains intact despite the bilateral involvement of the pons.

An 87-year-old male suddenly felt weakness in the legs at one night. When he woke up the next morning, he felt weakness in the arms as well. On that day he was admitted to the hospital. His past history was unremarkable. He had been a heavy smoker (20 tobaccos/day) until 5 years ago. He did not have a habit of drinking alcohol. On admission the blood pressure was extremely high (>200/150 mmHg). He complained of difficulties in moving the arms and legs. On the second hospital day he showed signs of bulbar palsy and tetraplegia. Extensor plantar response was bilaterally elicited. Brain MRI disclosed a “heart appearance” lesion in the pons (Figures 1(a) and 1(b)), which was high on diffusion-weighted image MRI (Figure 1(a)) and low on apparent diffusion coefficient map MRI (Figure 1(b)). Brain MRA demonstrated that the basilar artery remained intact (Figure 1(c)). Blood cell counts were normal. Routine blood chemistry, including sodium (139 mEq/L), was normal. Cerebrospinal fluid was normal except for increased protein (100 mg/dL). He did not have diabetes and hyperlipidemia. Serum vitamin B1 was normal. Atrial fibrillation was negative. He was diagnosed as having fresh, bilateral pontine infarction with a “heart appearance.” A treatment with ozagrel sodium, edaravone, and glyceol was initiated, which was later followed by oral aspirin. In spite of this treatment and rehabilitation, he remained tetraplegic; the condition of which was consistent with “locked-in syndrome.” He was transferred to another hospital for long-term care. 

## 3. Discussion

 “Heart appearance” on MRI was originally reported for bilateral medial medullary infarction [1–3]. In “heart appearance” infarction of the medulla, the anterior-medial territory and the anterior-lateral territory are involved, resulting in the “heart appearance” of the infarct [1, 2]. Since the scheme of vascularization of the pons is identical to that of the medulla [4], it should not be surprising to encounter “heart appearance” infarction of the pons. Kumral et al. [4] reported a comprehensive review of 150 cases of pontine infarction. They reported 14 cases (11%) of bilateral pontine infarction, which manifested with transient consciousness loss, tetraparesis, acute pseudobulbar palsy, or locked-in syndrome. The bilateral infarcts were schematically presented in this paper, and some of them were morphologically consistent with “heart appearance.” They postulated basilar artery atheroma, including basilar artery branch disease, or small artery disease as a cause of bilateral pontine infarction. The present case is another example of “heart appearance” infarction of the pons. It also suggests that bilateral ischemic involvement of the pons, regardless of whether it is due to basilar artery atheroma or small artery disease, is possible even in the context of an intact basilar artery. This is particularly important to fend off misleading diagnoses, such as central pontine myelinolysis. 

## 4. Conclusion

The present case is an example of “heart appearance” infarction of the pons that showed an intact basilar artery on MRA, suggesting that bilateral ischemic involvement of the pons is possible even in the context of an intact basilar artery.
